# Children’s susceptibility to content generated by artificial intelligence

**DOI:** 10.1016/j.techsoc.2026.103303

**Published:** 2026-03-18

**Authors:** Allison Langer, Steven Martinez, Peter J. Marshall, Jason Chein

**Affiliations:** Department of Psychology and Neuroscience, Temple University, PA, USA

**Keywords:** Generative AI, Children, Human-computer interaction, Developmental psychology

## Abstract

Advancements in artificial intelligence (AI) tools can make it difficult to discern what is “real”. Though children are interacting with AI-generated materials through entertainment and learning applications, no published work directly considers children’s ability to distinguish human-created from AI-generated information. In the current study, 37 children (6–10 years old) heard a narrative about a “SmartBot” (representing AI) that could write text, invent photos, and create art. They were then asked to discern which in a series of stimuli including short texts and images of objects, art, and faces, had come from the SmartBot vs. a human teacher and answer questions regarding their beliefs about AI. Children also completed measures of abstract reasoning and receptive vocabulary, and parents completed a home technology use survey. A separate sample of 49 adults (18–34 years) served as a comparison group for accuracy scores on the set of stimuli. On average, children performed at or below chance in discriminating human-created from AI-generated content across modalities, and their overall accuracy was significantly below that of adults. Children’s individual discernment varied and was significantly and negatively associated with parent reports of children’s at-home technology use. Children had varied beliefs about AI agency and experience, but broadly understood that an AI agent (the SmartBot) is not alive (95%). Our findings suggest that spending more time on digital devices is associated with susceptibility to artificial content and risk for misattributing AI content as human, highlighting the importance of early AI literacy to support children’s engagement with AI technologies.

## 1. Introduction

Advancements in artificial intelligence (AI) tools have made it increasingly difficult to discern what is “real”. Children are encountering AI in many contexts of their daily lives, through entertainment platforms, learning applications, and home devices like smart speakers. Children’s access to AI is also becoming increasingly widespread, with one survey showing that eighty-eight percent of children have access to, or own, a smart speaker, and forty-five percent of 6-to-9-year-olds speak to their smart speaker daily (Bickham et al., 2024). Surveys also show that children as young as 3 are using generative AI tools like ChatGPT—about half of parents of 3- to 12-year-olds report that their child has used generative AI, including for seeking information or advice and for creative activities such as storytelling or art (Bickham et al., 2024). Common Sense Media (2025) similarly finds that children ages 0 to 8 use generative AI to learn about school-related material, for critical thinking skills, or to create content like short stories or art (Mann et al., 2025). AI content that children hear from a speaker or consume online may not be explicitly labeled as such, making it important to understand how children perceive and evaluate AI-generated content.

Prior research examining how children conceptualize AI has largely focused on their beliefs about a technology’s capabilities and human-likeness. This work shows that children tend to anthropomorphize AI agents, but the degree to which they do so depends both on their age and on the type of device. Children understand that AI agents like Amazon’s Alexa or robots are not alive, yet they still may ascribe human-like characteristics to these technologies, like the ability to think, feel, and know things (Flanagan et al., 2023). In studies where elementary students were asked to draw what they think AI is, children frequently gave AI human-like appearances (Chang et al., 2026; Kalemkuş & Kalemkuş, 2025). When children encounter technologies that appear to possess human-like qualities—such as the ability to converse contingently or act on an environment—they are more likely to attribute agency to them (Flanagan et al., 2023; Oh et al., 2025), with younger kids having a greater tendency to overestimate agency compared to older kids (Andries & Robertson, 2023). Generative AI platforms are designed to produce content that mimics human-created content, raising questions about how children at different developmental stages will understand these systems’ capabilities and limits, and in turn how children evaluate and trust their content.

Children are generally skeptical of implausible claims (Browne & Woolley, 2004) and untrustworthy informants (Sobel & Finiasz, 2020). However, the strategies they use to detect what is plausible and trustworthy may not translate to online environments that are embedded with AI (Shtulman, 2024). Research on children’s experience in seeking information from the internet shows that preschoolers use a computer’s prior accuracy to determine whether to trust it (Danovitch & Alzahabi, 2013), and that, in some cases, 5 and 6 year old children preferred to seek out factual information from a person rather than the internet (Wang et al., 2019). While these studies point to a skepticism that children may apply to search engines like Google, it remains an open question as to whether children will apply the same amount of scrutiny to content derived from a generative AI platform that is designed to be convincingly human. While AI-generated content is not inherently false, children’s increased awareness of the source of content they encounter would enable children to apply the same critical evaluation to both human- and AI-generated information.

Empirical work shows that adults consistently perform near or slightly above chance when discerning AI-generated texts and images (Chein et al., 2024; Dugan et al., 2023; Miller et al., 2023) and that this discernment ability may be linked to factors such as fluid intelligence and time spent on digital devices (Chein et al., 2024). However, no study has investigated this ability in children, or whether individual characteristics make children better able to detect AI-generated content. As AI models continue to advance, the outputs they generate may become completely indistinguishable from human content for children and adults alike. Rather than focusing solely on detection accuracy—which may one day prove impossible—studying children’s discernment of AI content provides a window into their reasoning, trust, and mental models of AI, dimensions that will remain relevant even as AI technologies evolve. This gap motivates the current study, which examines children’s AI discernment across information modalities, factors that may support or hinder this ability, and children’s broader conceptualizations of AI compared to human-created content.

### 1.1. The current study

The present study investigates children’s ability to distinguish between content that is human- or AI-generated across multiple modalities, including text, objects, art, and faces. We further examine if this ability is associated with fluid intelligence, receptive vocabulary, age, or technology exposure. We assess children’s confidence in their discernment ability, the heuristics they use to make distinctions, and their conceptualizations of AI more broadly. Together, these analyses provide insight into children’s emerging AI literacy, and the factors that shape their evaluation of generative content.

## 2. Methods

### 2.1. Participants

Child participants (n = 37) ages 6 to 10 years old (mean_age_ = 7.87, SD_age_ = 1.11) completed the study remotely via Zoom. This age range was selected based on prior developmental work on child-technology interactions with similarly aged children that found meaningful trends in perceptions of AI (Oh et al., 2025) and machines broadly (Flanagan et al., 2023). Our sample size was determined by practical constraints of recruitment and data collection capacity during the study period, combined with power analyses suggesting this sample would provide adequate power (β = 0.80) to detect medium-to-large correlations. We acknowledge that the sample size is modest, particularly for detecting small effects.

Participants were recruited through a developmental psychology laboratory on a university campus in a small city in the eastern United States. The study was advertised through emails sent to mailing lists of families who had expressed interest in participating in research at the university. The study session lasted about 1 h. All research was performed in accordance with the regulations of the IRB at [blank] University, where the lead researchers acquired approval, and with the Declaration of Helsinki. Participants were compensated with a $10 gift card for their time.

Participants’ parents/legal guardians signed an online informed consent form prior to the Zoom session, and children gave verbal assent before participating in the task. Participants’ parents/legal guardians were asked to select all races/ethnicities they identified their child as being, and parents identified their child as: African/African American/Black (2), Asian/Asian American (2), Caucasian/European American/White (31), Hispanic/Latino/Latina/Latinx/Latine (1), Middle Eastern or North African (2), Biracial/Multiracial (4), did not respond (2). Parents/legal guardians described their family income as: $35,000 through $49,999 (2, 5.4%), $50,000 through $74,999 (1, 2.7%), $75,000 through $99,999 (5, 13.5%), $100,000 through $124,999 (9, 24.3%), Greater than $125,000 (18, 48.6%), did not respond (2, 5.4%)

We recruited and tested a sample of adults on our task stimuli to serve as a reference group for interpreting our child-specific findings. Adult participants were recruited on Prolific (www.prolific.com), a platform that facilitates recruitment for online research studies. Participants were required to be between 18 and 34 years of age, fluent in English, and located in the United States. In total, 50 participants were initially recruited, but one was excluded from analyses for not completing the full study sequence. The final sample included 49 participants (M age = 29.27 years, SD age = 3.79 years; 25 women, 24 men). All participants provided electronic informed consent, as approved by the Temple University Institutional Review Board. Participants were compensated via electronic payment at an hourly rate of $13.50 per hour.

### 2.2. Research design

#### 2.2.1. Human/AI discernment task

**Fig. 1.**
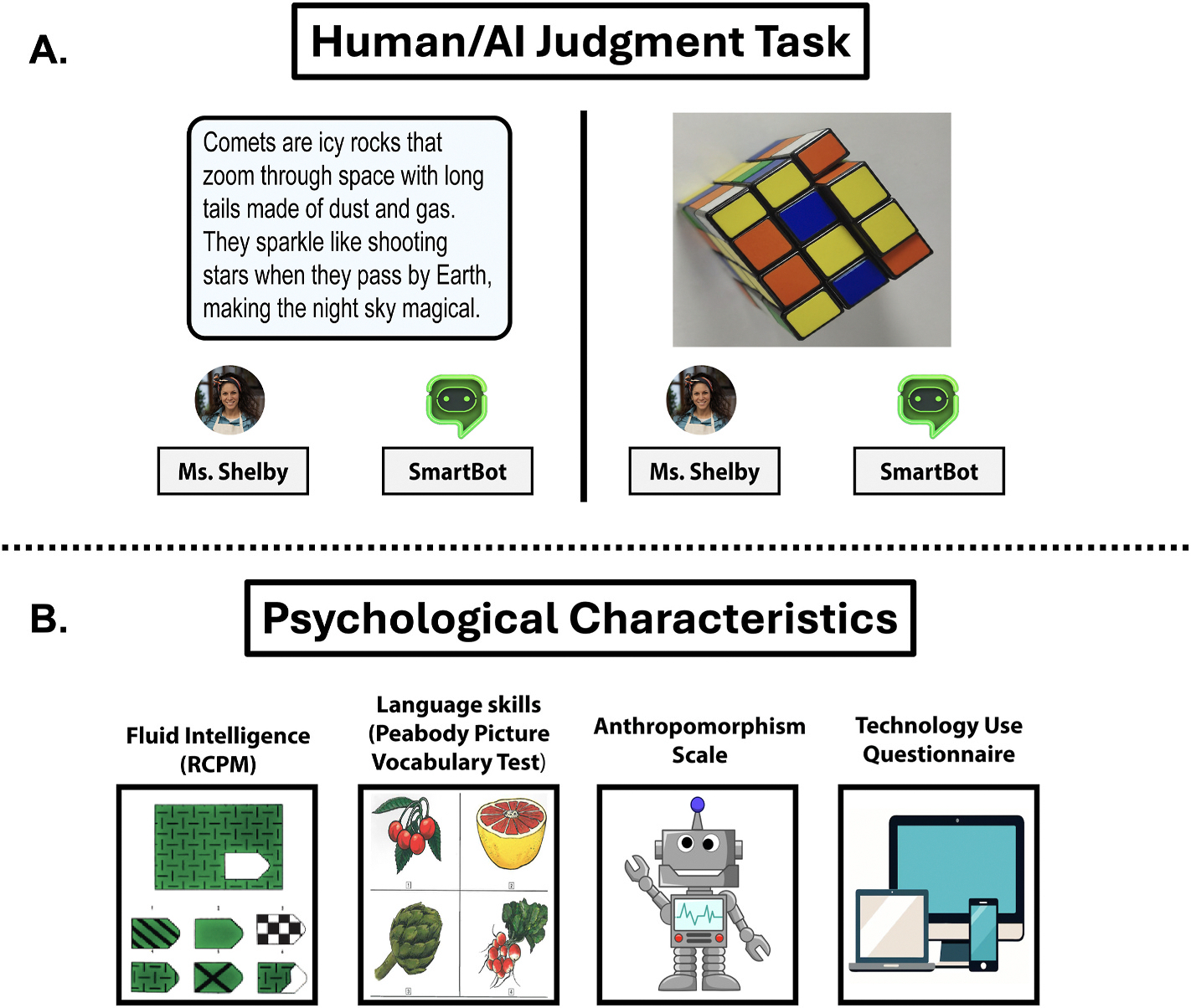
A. An example of the human/AI judgement task showing an example of a text stimulus (left) and image stimulus (right) and the options to choose either “Ms. Shelby” or the “SmartBot.” B. A depiction of the psychological characteristics and measures in the study, including the Ravens Colored Progressive Matrices (RCPM), the Peabody Picture Vocabulary Test, an Anthropomorphism Scale, and a parent report technology use questionnaire.

Children completed a human/AI discernment task implemented in jsPsych version 7.2.1 (see Fig. 1). At the start of the task, children were provided with an age-appropriate narrative illustrating how a “SmartBot” (representing an AI) and “Ms. Shelby” (representing a human), can each write text and create images. Children then engaged in an interactive demonstration where they were first shown examples of texts and images that came from Ms. Shelby and then were shown in real time how the SmartBot can create similar texts and images. For example, a short story about space was presented as having been written by Ms. Shelby, and then an experimenter entered a prompt into a text box telling the SmartBot to write a story about outer space. The SmartBot was shown to generate a short story about outer space. This demonstration was then repeated for images; an image of a golden retriever was presented as having been taken by Ms. Shelby, and in real time, children watched the experimenter enter a prompt into a text box telling the SmartBot to create an image of a golden retriever dog. The children then saw the SmartBot produce an AI-generated image that paralleled Ms. Shelby’s original photo. Children were then told that they were going to play a game where they would be asked to decide if they thought each in a series of stimuli came from the SmartBot or Ms. Shelby. Children received instructions on how to make their selections and completed four practice rounds before the task began. Children were informed that exactly half of the stimuli came from Ms. Shelby, and half came from the SmartBot.

#### 2.2.2. Human-created and AI-generated texts and images

One-hundred twenty-eight total stimuli were included in the Human/AI Discernment Task across four categories: texts (32), and images of art (32), objects (32), and faces (32). To ensure that variation in image size did not bias human/AI judgments, all task images were shown in the same size (1212 × 1082 pixels). Human texts were taken from non-fiction children’s books from the website “epic!” (https://www.getepic.com/), a digital repository of children’s books. Short, 30–35-word blurbs were taken from 32 texts, selected based on reading level (suitable for 6–10-year-olds). The topics of texts related to animals, outer space, seasons, transportation, and jobs. Thirty-two matching AI-generated texts were created using ChatGPT (version 4.0) with prompts that were designed to produce outputs that paralleled the human texts regarding their subject matter and their approximate word count. For example, given a human text about the moon, the following prompt was entered into ChatGPT to generate a parallel text: “write 30–35 words about the moon that are suitable for 6–10-year-old children to read.” After 32 human texts were sourced and 32 AI-generated texts were generated, 16 human were randomly selected to be included in the task. After the first 16 human texts were randomly identified to be included in the task, the non-matching 16 AI tasks were also selected, such that there were 32 unique subjects for texts across the task (i.e. if the human text about a moon was selected, the AI-generated text about the moon was discarded). To account for varying reading levels among participants in our sample, we used a text-to-speech reader to create audio of each text stimulus that were played simultaneously as each text was presented in the task. The same voice was used across the entire task, for both human and AI-generated texts.

Human images of objects were sourced from stock and publicly available images on Flickr (https://www.flickr.com/). Images were selected if their upload date preceded 2017 to reduce the likelihood of inadvertently selecting AI-generated images. Images of objects were selected around child-friendly scenes, including amusement parks, picnics, and items you may find in a school. AI-generated images were then generated using Google Gemini 1.0 with prompts that were designed to produce outputs that matched the human object images. For example, if a photo of a popsicle was selected from Flickr (pre-dating 2017), the following prompt was entered into Gemini to generate a matching photo: “create a photo of a popsicle.” After 32 human object images were sourced and 32 AI object images were generated, 16 human-generated object images were randomly selected to be included in the task. After the first 16 human images of objects were randomly identified to be included in the task, the non-matching 16 AI images of objects were also selected, such that there were 32 unique object images across the task.

Human images of art were sourced from public domain art sites, like wikiart.org. Artwork was selected if deemed child-appropriate by the research team and included paintings and sketches of various scenes (e.g., fruit bowl, flower bouquets, paintings of a cat). All the selected artwork was created by known human artists (e.g., Van Gogh). AI-generated art images were then generated using Google Gemini 1.0 with prompts that were designed to produce outputs that matched the human art images. For example, if a painting of a farm was sourced, the following prompt was entered into Gemini to generate a matching photo: “create a painting of a farm.” After 32 human art images were sourced and 32 AI art images were generated, 16 human-created art images were randomly selected to be included in the task. After the first 16 human images of art were randomly identified to be included in the task, the non-matching 16 AI images of art were also selected, such that there were 32 unique art images across the task.

Human and AI-generated images of faces were sourced from Nightingale and Farid (2022), which contains 400 human faces and 400 AI-generated faces of both children and adults (Nightingale & Farid, 2022). Nightingale and Farid (2022) used 400 human face images from the Flickr-Faces-HQ (FFHQ) dataset to create 400 matching AI-generated face images using a StyleGAN2 neural network model. We randomly selected 16 human face images and 16 non-matching AI-generated face images from this data set and ensured equal distribution across gender and race.

#### 2.2.3. Confidence judgments & open-ended responses

After child participants completed the discernment task, they were asked to judge their confidence in their choices. For each stimulus category (images of objects, art, and faces, and texts) and source type (human or AI), a representative example was shown, and children were reminded of the choice they had made earlier in the task for that stimulus (“You said that this [stimulus] came from the SmartBot/Ms. Shelby). They were then asked, “How sure are you that this [stimulus] came from the SmartBot/Ms. Shelby?” with Likert choices ranging from “not sure at all”, to “very sure”. Then, they were asked in an open-ended format to describe why they thought the stimulus came from the SmartBot/Ms. Shelby. In total, children made 8 confidence judgments and gave 8 open ended descriptions of their judgements.

To systematically analyze the reasoning strategies children employed when making human/AI judgments, we developed a comprehensive coding scheme for children’s open-ended explanations. Responses were analyzed using computational text analysis methods implemented in the R package *tidytext* (Silge & Robinson, 2016). After reviewing children’s responses, we developed a coding scheme comprising seven heuristic categories: (1) visual quality cues (references to perceptual features such as blur or detail), (2) realism judgments (references to real or fakeness), (3) content plausibility (evaluations of logical consistency or factual accuracy), (4) knowledge of AI (explicit mentions of AI), (5) anthropomorphic reasoning (references to agency or intentions), (6) stimulus-specific references (mentions of particular features specific to a stimulus), and (7) uncertainty (expressions of being unsure or not knowing).

We created two complementary dictionaries to identify these heuristics: a single-word dictionary containing 48 keywords (e.g., “blurry”) and a multi-word phrase dictionary containing 138 expressions (e.g., “background is blurry”). Using *tidytext*, all responses were tokenized into individual words and searched for multi-word phrases. Common stop words (e.g., “the,” “a,” “is”) were removed from single-word tokens. Each tokenized word and phrase was then matched against the dictionaries to determine the presence (1) or absence (0) of each heuristic category. Words and phrases could match multiple heuristic categories within a single response. We calculated the frequency with which each heuristic type appeared across all responses, examined whether heuristic use differed by the content source (AI vs. human). For our full coding scheme and word dictionaries, please see our supplementary materials.

### 2.3. Psychological characteristics

#### 2.3.1. Raven’s Colored Progressive Matrices (RCPM)

Children’s individual differences in nonverbal fluid intelligence and abstract reasoning were assessed using the Raven’s Colored Progressive Matrices (Raven et al., 1995). The RCPM are designed for children aged 5 through 11 and maintains three sets of 12 items. Overall task performance is measured by the number of correct responses out of the 36 items. Prior work has shown that adults’ scores on the abbreviated version of the Raven’s Standard Progressive Matrices (Bilker et al., 2012) is related to their discernment of AI-generated texts (Chein et al., 2024); we sought to test this relationship in children.

#### 2.3.2. The Peabody Picture Vocabulary Test (PPVT-5)

Receptive vocabulary–child participants’ ability to understand spoken words—was measured using the Peabody Picture Vocabulary Test, 5th Edition (Dunn, 2019). The PPVT-5 consists of up to 240 items. A baseline score is established after a child answers three consecutive items correctly, and a maximum is determined after the child answers six consecutive items incorrectly. A total raw score is calculated from the total number of items children answered correctly. The PPVT has been linked to reading comprehension for elementary school children (Verhoeven et al., 2008) which may support recognition of AI-generated writing.

#### 2.3.3. Anthropomorphism scale

To index the degree of human-like qualities children attribute to the SmartBot, an anthropomorphism scale was developed, modeled after questions from Severson and Lemm (2016) (Severson & Lemm, 2016). The children were asked 8 yes or no questions about both the SmartBot and Ms. Shelby, which can be divided into questions related to “having experience” (*Does the agent have feelings? Is the agent alive? Can the agent feel offended? Does the agent have friends?),* and “having agency” (*Does agent choose to do things? Does the agent always give you correct information? Does the agent make decisions? Does the agent think for itself?).* Endorsements of “yes” were coded as a 1, and endorsements of “no” were coded as a 0. Cumulative agency and experience averages were derived from adding “yes” and “no” endorsements for each subscale and dividing by the number of questions (4).

#### 2.3.4. Technology use questionnaire

To assess children’s technology use at home, parents/guardians were asked to indicate the types of technology they have in their home (television, tablet or iPad, desktop computer, laptop computer, smartphone, video game devices, Amazon Alexa, Google Home, or other voice assistants, robot toys, other types of robots (i.e., robotic vacuum cleaners), and how often their child has used each type of technology in the past 7 days, from minimal or no use (0–2 times per week, coded as “0”), moderate use (3–6 times per week, coded as “1”), or frequent use (more than 6 times per week, coded as “2”). Cumulative technology frequency use scores were calculated by adding the counts of the frequency of use across all technology types. For example, if a child used an iPad moderately (1), and a television minimally (0), and Alexa frequently (2), they would receive a technology use score of 3. Recent work with adults (Chein et al., 2024) found that greater time spent online was associated with worse AI discernment, which we sought to test in children.

#### 2.3.5. Adult comparison sample

To be able to compare and validate our results from the child sample, we had adult participants complete the same human/AI discernment task. Adults were presented with an instruction explaining that the experiment was originally designed to test children’s ability to discern whether information was created by humans or generated by artificial intelligence, and includes child-friendly narratives, images, and instructions. Adult participants then completed an identical task, using identical stimuli to the child cohort. Here, we report only on adults’ accuracy on the task relative to children.

## 3. Results

### 3.1. Discernment accuracy

#### 3.1.1. Discernment accuracy between child and adult samples

**Fig. 2.**
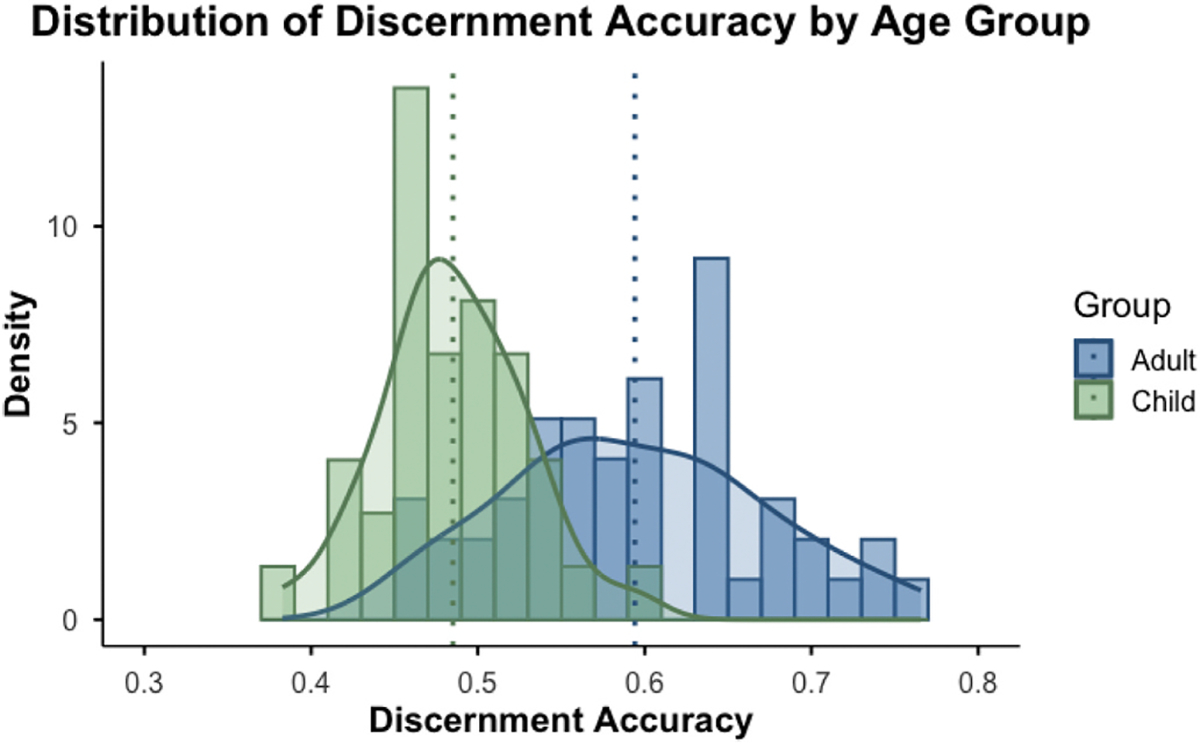
Histogram reflecting the distribution of overall accuracy scores on the human/AI discernment task between adult and child groups.

To first obtain a reference group for accuracy in discernment of our stimulus selection, we compared how adults performed relative to children in overall discernment. Given different sample sizes between our child and adult groups, a Welch two-sample *t*-test was conducted to examine differences in accuracy between the two groups. Results indicated that adults (M = 0.59) demonstrated significantly higher accuracy than children (M = 0.49), *t*(78.19) = 8.36, *p* < .001, 95% CI [0.08, 0.14] (see Fig. 2). This finding suggests a reliable difference in accuracy between children and adults. Additional graphs related to adults’ performance on the task can be found in our supplementary materials. The remaining analyses reflect only the child sample.

#### 3.1.2. Children’s discernment accuracy overall and by stimulus category

We analyzed children’s category-based detection accuracy. We calculated d’ signal sensitivity scores (Swets et al., 1961), where we defined a “hit” as correctly identifying human-created content as human-created, and a “false alarm” as mistakenly identifying AI-generated content as human-created. The average d’ sensitivity score for all stimuli was −0.077 (SD = 0.22). Since participants’ accuracy was below chance (50%), and there was an overall negative d’ sensitivity score, we conducted one-tailed t tests to see if there was systematic bias in our participants’ discernment choices. A one-tailed *t*-test revealed that participants were significantly below chance in their overall discernment accuracy (t(36) = −2.12, *p* = .02), and systematically over-endorsed stimuli as human rather than AI (t(36) = −2.15, *p* = .02).

**Fig. 3.**
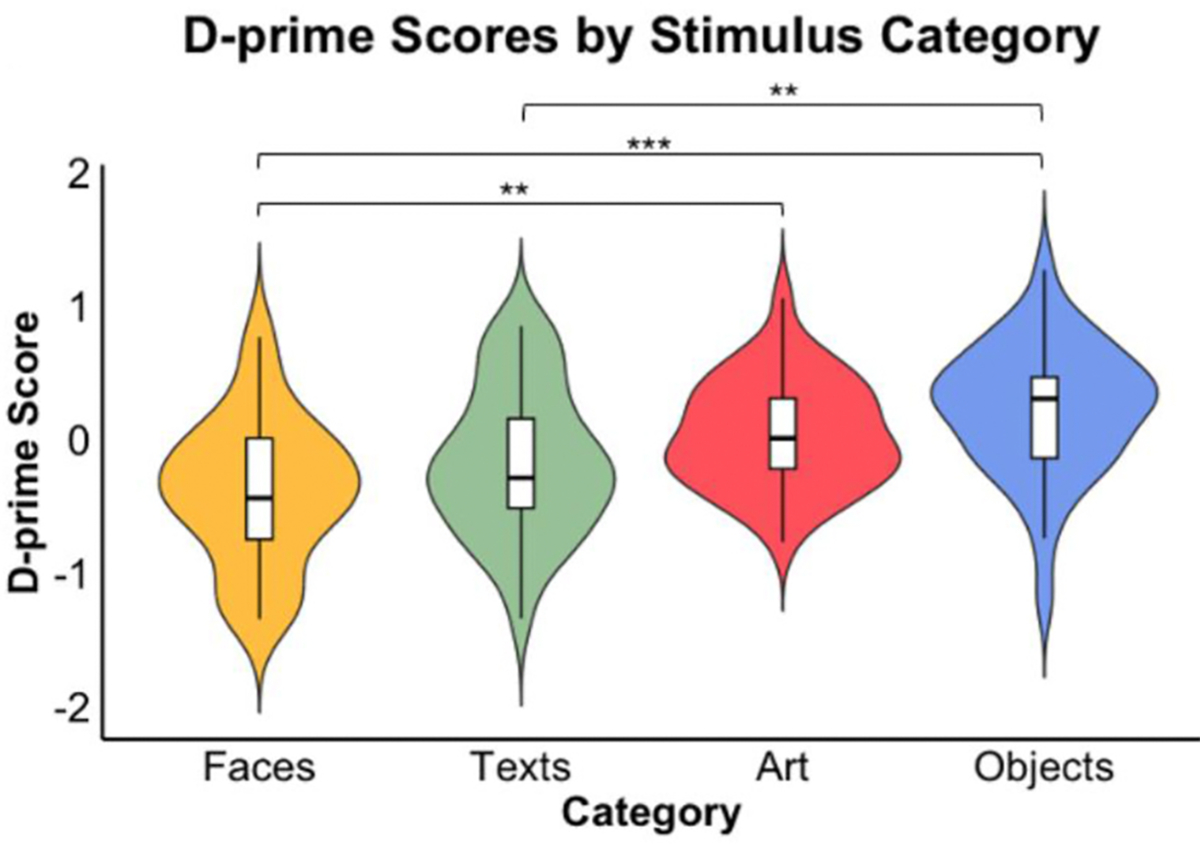
Violin plots showing D′ sensitivity scores by stimulus category.

Average accuracy and d’ sensitivity scores differed across stimulus categories (see Fig. 3). Average accuracy was highest for objects (M = 54%, SD = 19%), then art (M = 51%, SD = 18%), texts (M = 47%, SD = 19%), and faces (M = 43%, SD = 18%). Participants were significantly better than chance at discerning objects, t(36) = 2.70, *p* = .005, but significantly below chance for faces, t(36) = −4.62, *p* < .001, and texts, t (36) = −2.01, p = .03. Discernment accuracy for art was not statistically different from chance. A one-way repeated-measures ANOVA demonstrated a significant effect of stimulus category on d’ scores, F(3, 108) = 10.03, *p* < .001. Pairwise comparisons of d’ scores were significant for art and faces (*p* = .01), faces and objects (*p* < .001), and objects and texts (*p* = .02). For examples of the stimuli that were judged most and least accurately by participants, see our supplementary material.

### 3.2. Predictors of children’s discernment accuracy

**Fig. 4.**
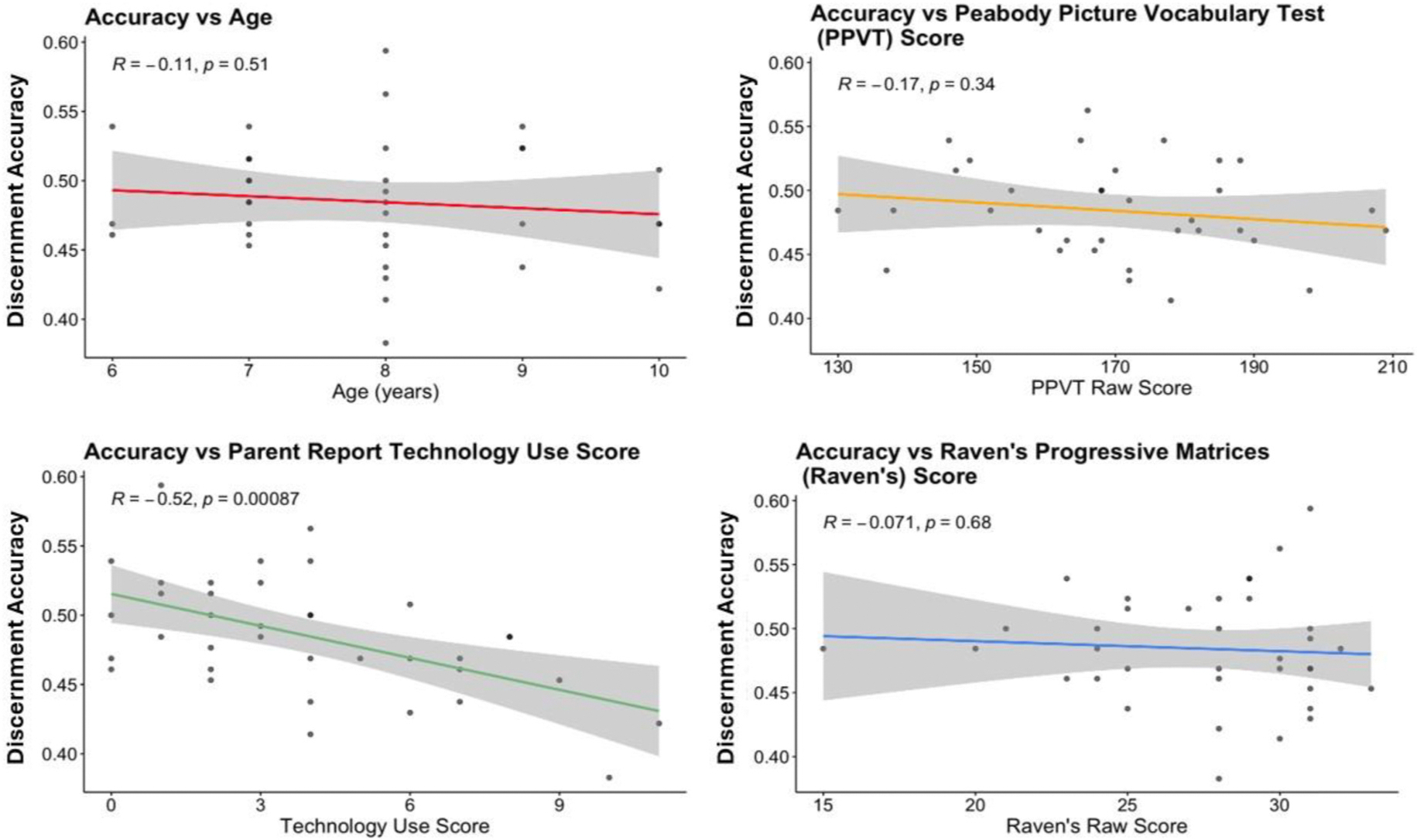
Scatterplots show how age and psychological characteristics relate to discernment accuracy.

We next examined how individual predictors account for variance in detection ability and children’s confidence in their judgments. Age, scores on the Raven’s Colored Progressive Matrices (RCPM), and scores on the Peabody Picture Vocabulary Test (PPVT) were not significantly correlated with detection accuracy (see Fig. 4). Parent-report technology use was significantly negatively correlated with detection accuracy, R = −0.52, *p* < .001, 95% confidence interval [−0.73, −0.24].

**Fig. 5.**
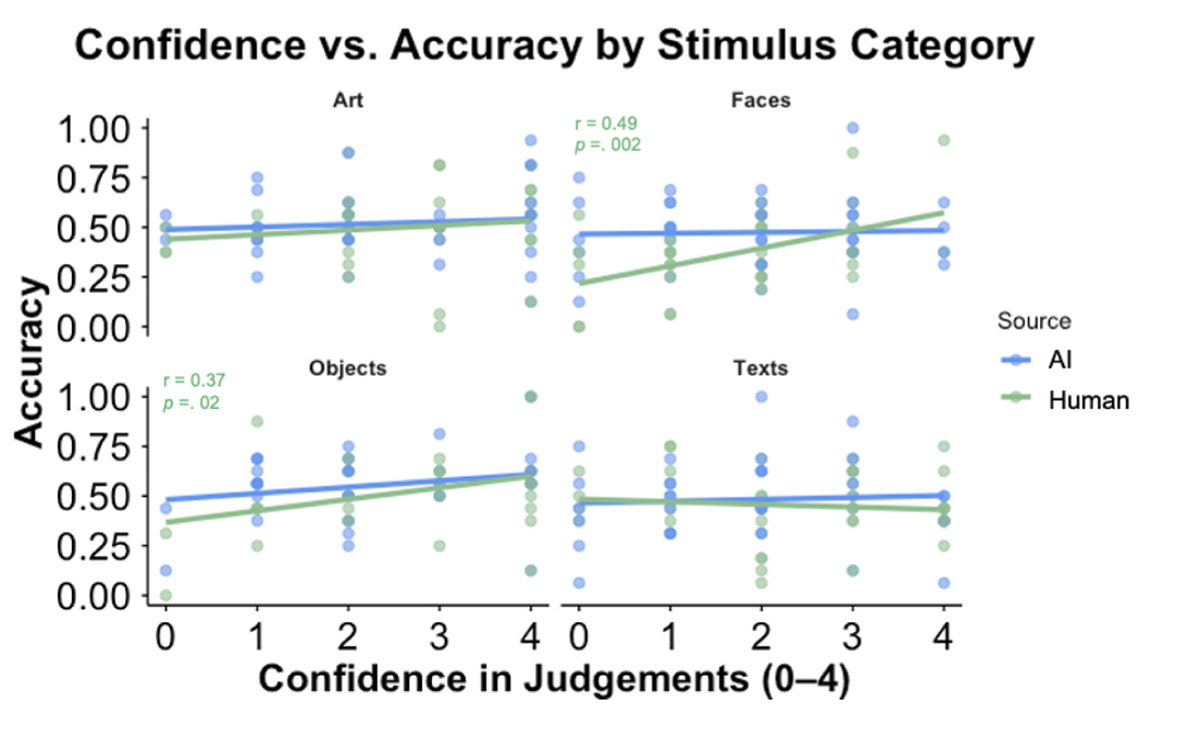
Scatterplots show children’s confidence in their judgements by stimulus category and source type.

Children’s confidence in their judgments was only significantly related to their accuracy for human generated objects (r = 0.37, *p* = .02) and human generated faces (r = 0.49, *p* = .002) (see Fig. 5).

### 3.3. Children’s perceptions of the SmartBot

Most children said the SmartBot was not alive (94.6%). However, around half of children said the SmartBot could make decisions (48.6%), think for itself (48.6%), and choose to do things (51.4%). Few children thought the SmartBot always gives correct information (21.6%) or has feelings (21.6%). Endorsing more anthropomorphic qualities to the SmartBot was not significantly related to age (r = −0.19, *p* = .26) or accuracy on the discernment task (r = −0.28, *p* = .09).

**Fig. 6.**
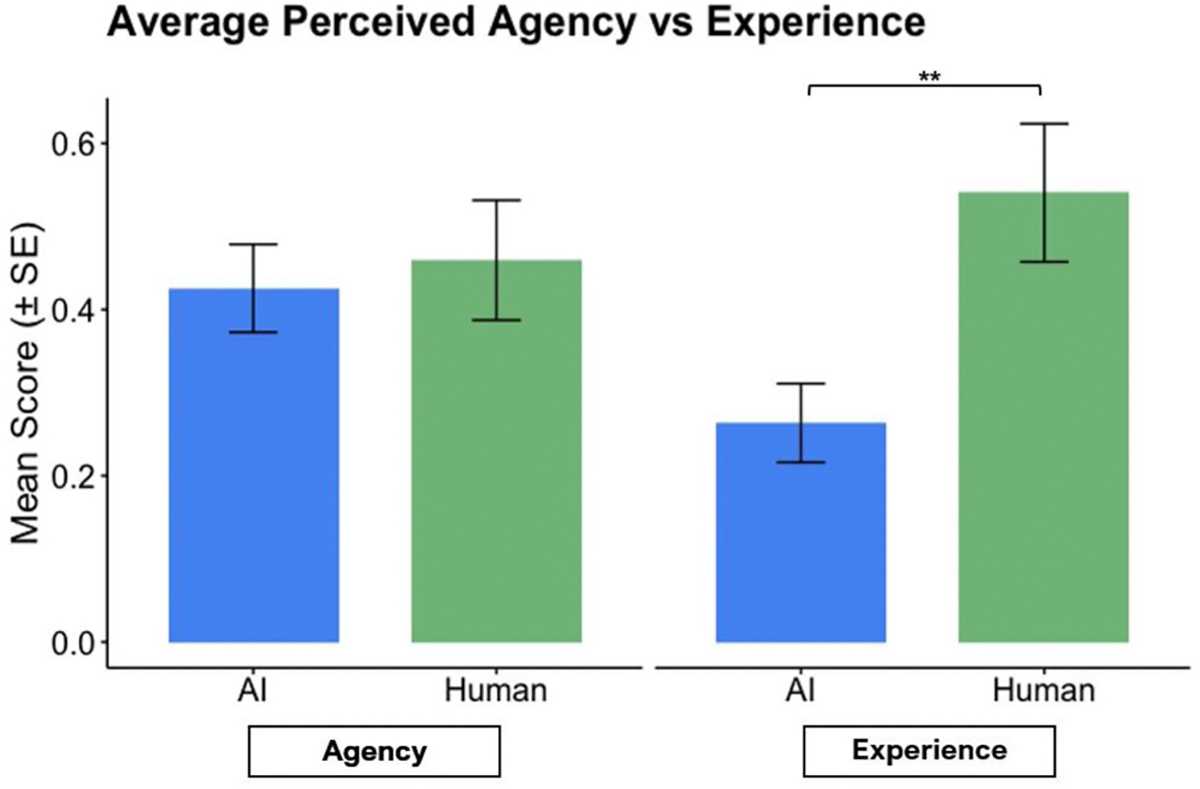
Bar plots showing participants’ average ratings for AI and human agency and experience.

A Welch’s two-sample *t*-test showed that there was no significant difference in perceived agency between the AI condition (M = 0.43) and the human condition (M = 0.46), t(66.04) = −0.38, *p* = .71. In contrast, participants attributed significantly more experience to humans (M = 0.54) than AI (M = 0.26), t(57.20) = −2.90, *p* = .005 (see Fig. 6). Participants’ age was not significantly related to perception of AI agency (*p* = .69) or AI experience (*p* = .13).

**Fig. 7.**
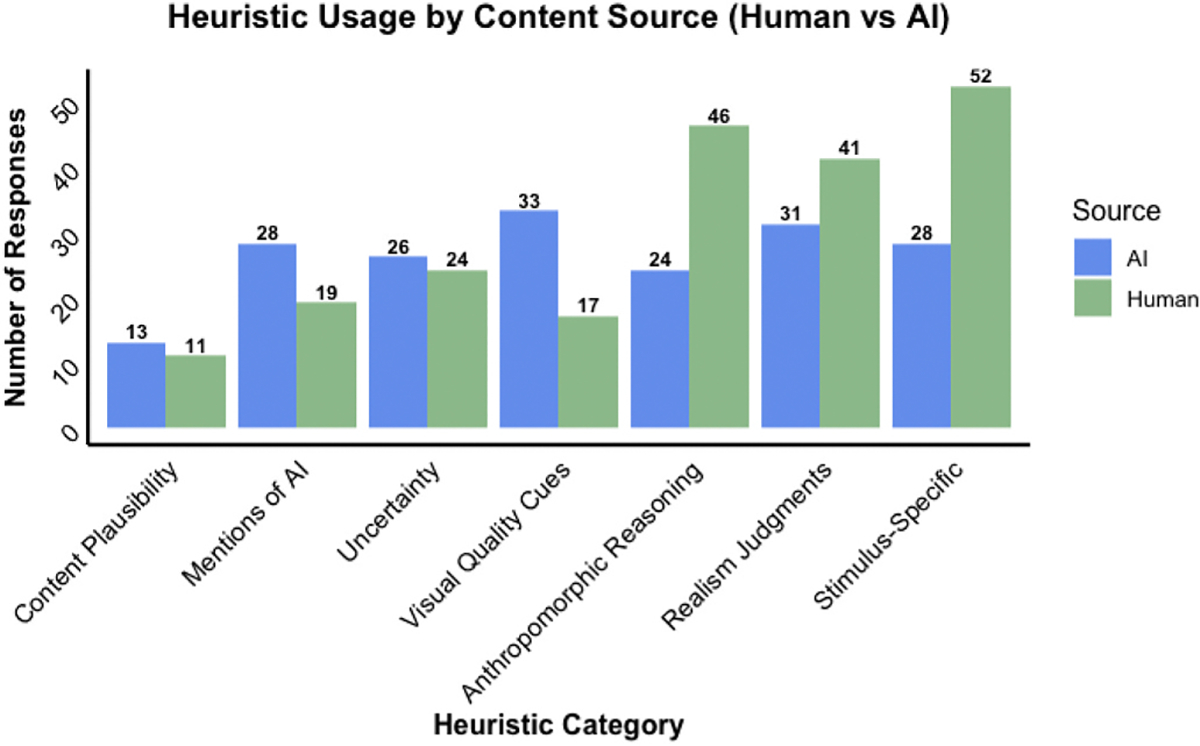
Heuristic categories children used by type of content they were judging (human or AI).

Children’s open-ended explanations revealed diverse reasoning strategies for judging whether content was human- or AI-generated. The most frequently employed heuristics were stimulus-specific references (80 mentions), followed by realism judgments (72 mentions), anthropomorphic reasoning (70 mentions), visual quality cues (50 mentions), uncertainty expressions (50 mentions), mentions of AI (47 mentions), and content plausibility (24 mentions). Fig. 7 shows the frequency with which each heuristic category appeared across responses by the true content’s source (human or AI), and Table 1 provides representative quotes for each heuristic category.

## 4. Discussion

We examined children’s ability to detect whether texts and images were AI- or human-created and found that children were generally unable to discern the source of the content. Children’s frequency of technology use at home was negatively related to their overall discernment accuracy. We found that children were slightly better at detecting objects and artwork but struggled to determine the source of faces and texts. D′ sensitivity scores demonstrated that children had a systematic bias towards perceiving faces as human, irrespective of their true source. Children’s open-ended responses regarding the heuristics they used to make their judgments revealed varying levels of familiarity with generated content and showcased the emphasis on what they considered to be “real” in their decision making. We discuss the implications of our findings for early AI literacy and the risk of children’s manipulation online.

### 4.1. Discernment accuracy

Overall, children struggled to discern whether content was human or artificially generated. We saw significant discrepancies in discernment across our stimulus categories, whereby children were most accurate in detecting objects and least accurate at detecting faces to be human or AI-generated. Object recognition may have been high due to the ubiquity and ecological relevance of objects in children’s everyday lives. In a sense, children may be “domain experts” in objects; the adult literature on AI discernment shows that in some cases, domain experts have higher accuracy at discerning content relevant to their expertise (Hitsuwari et al., 2023; Ma et al., 2023).

Children’s poor performance with faces likely reflects a different phenomenon: a systematic bias toward perceiving content as human created. Prior work with adults has shown that AI faces are indistinguishable from human faces (Nightingale & Farid, 2022) and that adults also have the tendency to overidentify AI faces as human (Miller et al., 2023; Shen et al., 2021; Tucciarelli et al., 2022). In the adult AI face discernment literature, people’s perception of real faces, rather than the realness of the faces themselves, predicted conformity and trust in the faces (Tucciarelli et al., 2022). Children’s inability to distinguish human and AI-generated faces, and their overattributing of AI faces as human, has important consequences for the potential of manipulation of children online.

### 4.2. Predictors of discernment accuracy

Despite the relatively wide developmental period covered by our participant sample (ranging from 6-to-10-year old’s), we did not find that age was predictive of discernment accuracy. Given that adults struggle to accurately distinguish realistic AI-generated images and texts from human-created content (Chein et al., 2024; Dugan et al., 2023; Miller et al., 2023), this finding is not surprising. Discernment accuracy was also not related to performance on our receptive vocabulary or fluid intelligence and abstract reasoning tasks. AI-discernment in adults has been positively linked to abstract reasoning skills (Chein et al., 2024), but we did not replicate this result in our younger sample. This null finding may reflect floor effects in discernment accuracy that limited variability, or a developmental difference whereby the cognitive processes supporting AI discernment in adults have not yet emerged in middle childhood.

However, we did find that parent-report technology use was negatively related to children’s accuracy on the discernment task. Children who spent more time using technology at home, including smartphones, tablets, and voice assistants, tended to perform worse on the AI-discernment tasks. This finding mirrors results from AI discernment studies with adults finding that spending more time online exacerbates the misattribution of AI-generated outputs as having human origin (Chein et al., 2024). One explanation for these findings may be that heavy technology users may be exposed to more AI-generated content, which may normalize AI outputs for users and reduce their sensitivity for detecting subtle signals that differentiate human and AI content. Technology use may foster familiarity without critical discernment, which highlights the need for nuanced AI literacy programs for children. Future work may better delineate which specific types of technology engagement (social media use vs. direct use of AI) are most predictive of detection ability, and whether training interventions can improve detection.

The negative association between parent-report technology use and children’s accuracy on the discernment task may alternatively be explained by considering how exposure to AI content may shape children’s heuristics. Children who use digital devices more frequently may have more exposure to more advanced AI models (ChatGPT-4o or Gemini 2.0), whose outputs tend be hyper-realistic. This familiarity may lead children to conceptualize AI content as *more* polished than human content, which may inadvertently lower accuracy on our task which generated stimuli from less current, and more flawed AI models (ChatGPT-4 and Gemini 1.0). If true, future work that uses more advanced AI models may see that children’s exposure to AI, via more frequent technology use, may relate to better discernment.

Children were overall mildly confident in their discernment judgments. Their confidence in judging stimuli to be human- or AI-generated was only related to their accuracy in their judgements for human-generated faces and objects. This may suggest that children have some awareness of their ability to reliably detect specific information—in this case from human sources—but their confidence does not consistently align with accurate decision-making. This finding has important implications for AI literacy education. Rather than focusing primarily on teaching children to detect AI-generated content—a skill that may become increasingly unreliable as AI advances—AI literacy interventions may need to emphasize broader critical thinking strategies, including how to evaluate information quality, seek verification from multiple sources, and recognize that confidence or intuition may not suffice for assessment of AI content.

While children were largely not sure of their decision making, and their decisions were not accurate themselves, children still used heuristics to make their decisions on the source of the content they viewed (see Table 1 with quotes). When prompted to explain their judgments, children tended to focus on specific features of the stimulus they were presented, though they also gave more generalizable strategies for detecting AI from human-created content more broadly. Children emphasized how “real” images looked or texts sounded and tended to attribute “real”-ness with human rather than AI-generated content.

Using anthropomorphic reasoning, such as referencing what Ms. Shelby or the SmartBot could or could not do, was also a common approach. Children referenced visual features both at the stimulus-specific level (e.g., “the corn, and how it’s burnt, all of the details—only a photo could capture that, the SmartBot couldn’t capture all of that detail”) and as general detection strategies (e.g., “sometimes generated images are blurry in the background”). Notably, though the task never used the phrase “artificial intelligence,” and instead referenced the “SmartBot” exclusively, children frequently mentioned “AI” and “AI-generated” in their open-ended responses. These results demonstrate that young children are aware of AI-generated material and concepts, though they may not recognize the extent to which this material can mirror “real” or human-created content.

### 4.3. Perceptions of the SmartBot

We found that children ascribed a similar degree of agency to the SmartBot and Ms. Shelby, like their ability to think for themselves or make decisions. However, children ascribed significantly more ability to experience things to Ms. Shelby relative to the SmartBot. This pattern aligns with prior work on children’s perception of AI-enabled smart speakers, where 4–8-year-old children rated a smart speaker as having similar levels of agency but less experience than a physically present human (Oh et al., 2025). Interestingly, age was not related to perception of agency or experience attributed to the SmartBot in our study, despite prior findings showing an association between age and beliefs about machines (Mann et al., 2025; Xu et al., 2025). Though the extent to which children attribute agent-like features to technology may typically decline with age (Mann et al., 2025; Xu et al., 2025), such findings have focused on technology that children can interact with (i.e., technology with a physical presence). The virtual (i.e. non-physical) nature of generative AI may represent a different form of technology that further blurs the line between agent and human in ways that could attenuate or delay the age-related decline in anthropomorphism observed with other technologies.

### 4.4. Limitations

While our study provides novel insights into how children perceive content generated by AI, our findings are limited in part by a small sample size. Further, our participant pool was recruited solely from a small city in the eastern United States, with high average annual income and caregiver education levels. While such results are still informative, they may not generalize to children in different geographic or socioeconomic contexts with different exposure to, experience with, or beliefs about AI.

During study design, we also had to make challenging decisions about how to introduce the AI agent and explain what it can do, while limiting any biasing effects. Given the young age of the participant pool, we decided to expose children to our AI condition via a narrative about the “SmartBot” and its capabilities to produce images and texts. Though our demonstration of the SmartBot’s image and text generation aimed to provide children with an accurate representation of the capabilities of the generators we used to source our stimuli, this narrative may have influenced participants’ perceptions about the SmartBot in ways that do not generalize to AI more broadly. Future work that explicitly names AI as “AI” may better capture children’s broad perceptions of the technology. Additionally, while the images and texts used in the demonstration portion of the experiment were created from the same AI models as the task stimuli, and therefore should have similar levels of AI artifacts or signals (e.g. blurriness, pixelated images), it is possible that the selection of stimuli used in our demonstration and practice rounds somewhat influenced children’s decisions in the task itself. Future study designs can randomize all stimuli across the demonstration, practice rounds, and the task to minimize the risk of priming effects.

Further, we told participants that half of the stimuli they would see came from the SmartBot, while half of the stimuli came from Ms. Shelby. It is possible that a child who thinks that all information they encounter on the internet comes from a human may have only selected SmartBot *because* of this instruction. Importantly, children showed a human-attribution bias despite being explicitly told that exactly half the stimuli were AI-generated. This suggests a bias for human attribution that persists even with instructions that direct children otherwise, though the magnitude of this effect may have been stronger had we not provided information of the base rate of the sources of the stimuli. Thus, the human-attribution bias we observe may underestimate the magnitude of children’s bias in real-world contexts where AI content is often unlabeled.

The AI models we used to generate our stimuli, including ChatGPT 4.0, Gemini 1.0, and the generative adversarial networks (GANs) (Nightingale & Farid, 2022), differed across our stimulus categories. The differences in AI generators likely contributed somewhat to the observed category differences (e.g., difficulty with objects vs. faces), but they may also limit the interpretability of absolute accuracy scores. Further, new updates to these models have improved the quality of images to reduce artifacts that children may have observed, such as “blurry backgrounds” or “pixelated” images. Our results should be interpreted in the context of our stimuli set, but may not generalize to future AI models or across different AI generators.

We found that children’s cumulative time spent using various devices at home was negatively related to their discernment of AI stimuli. While this finding is robust in our sample, it is still important to exercise caution when interpreting this result. The 95% confidence interval for this correlation is [−0.72, −0.24], indicating uncertainty about the precise magnitude of the effect, and replication with larger samples can help establish the stability of this effect. Our parent-report technology use survey asked if children had interacted with generative AI, and parents unanimously endorsed the “no” response, suggesting that the parents may be underestimating the range of contexts in which generative-AI functionality is already embedded. Our open-ended responses regarding the heuristics children used to make their judgements suggest that children are aware of generative AI to some extent. Future research might aim to more aptly capture children’s prior exposure to AI, so we can clarify which types of technological engagement are most predictive of children’s discernment and conceptualizes of AI-generated content. Even so, the observed link between broad technology use and reduced discernment suggests that digital experience may play a role in how children evaluate AI-generated content; a finding that resonates with observations made in adult samples (Chein et al., 2024).

The present study offers no direct assessment of how AI discernment might change with age. Within the relatively narrow age band assessed (6–10-year-olds) we saw no indication that AI-discernment skill improved alongside cognitive development (e.g., there was no correlation between participant age and discernment task performance). While there are some challenges in designing a study of age-dependent change in AI discernment, a future study including cross-sectional age cohorts, or tracking this ability longitudinally, could provide a clearer characterization of the cognitive and experiential factors that affect discernment skill.

## 5. Implications

Our finding that children cannot discern whether content is human or AI-generated has several implications for how children navigate their online and everyday environments. Poor discernment may make children especially susceptible to misinformation or manipulation (Yu et al., 2025), particularly when viewing “deepfakes,” or images of people that they believe to be real. Further, over-attributing AI content as human-created may lead children to trust it more than warranted. AI watermarking and transparency, where platforms are required to label AI-generated content as such or maintain clear distinctions between AI’s capabilities and human-like traits (Collyer-Hoar & Rubegni, 2025; Kurian, 2025), may reduce the risk that children are misled by non-human generated content. These findings highlight the urgent need for AI literacy tools that equip children and families with skills to critically evaluate the capabilities and limitations of AI devices. Further, the findings call for more longitudinal, developmental research tracking how children’s perceptions and understanding of AI change with age and exposure.

## 6. Conclusion

Though children used heuristics to make discernments, they were generally unable to detect AI-generated content from human-created content. Detection accuracy varied by content type; objects and art were slightly easier to detect than faces and texts. Negative D′ values suggest that children have a bias towards perceiving content as originating from humans. More frequent technology use was related to poorer discernment accuracy, suggesting spending more time on devices may lead to susceptibility to artificial content and risk for misattributing AI content as human. Our findings highlight the importance of early AI literacy to support children’s engagement with AI technologies.

## Supplementary Material

MMC1

## Figures and Tables

**Table 1:** Representative participant quotes regarding their judgments of stimuli in relation to heuristic categories.

“Can you tell me why you think this came from the SmartBot/Ms. Shelby?” Representative Participant Quotes		

Heuristic Category	AI-Generated Content	Human-Created Content

Stimulus-Specific	*“Real picture would have shadows and if you look at the background it looks yellow-ish and the sky is blue not yellow”*	*“Ms. Shelby works at school and maybe she took pictures of lockers”*
Realism Judgements	*“It looks unreal”*	*“Because it’s real, it’s looks very very real”*
Anthropomorphic Reasoning	*“Most of the texts that I choose the SmartBot for related the objects to magic, most people wouldn’t do that, don’t believe in magic”*	*“She’s creative and maybe she likes butterflies”*
Visual Quality Ques	*“Because the photo is stretched out, and a photo from a regular camera would be straightened”*	*“Because there are a lot of details”*
Uncertainty	*“Took a random guess on this one”*	*“I’m not sure”*
Mentions of AI	*“Because it kinda looks AI-generated”*	*“SmartBot was doing shorter paragraphs, this is longer than what AI would do”*
Content Plausibility	*“If it came from the SmartBot it could have anything on it”*	*“Because it is true”*

## Data Availability

Data will be made available on request.

## Appendix A. Supplementary data

Supplementary data to this article can be found online at https://doi.org/10.1016/j.techsoc.2026.103303.
