# Supplementary material for: Children’s susceptibility to content generated by artificial intelligence
Source: Technol Soc. Author manuscript; Available in PMC 2026 Apr 21. (PMC13089802; doi:10.1016/j.techsoc.2026.103303)
Supplement: MMC1 [file NIHMS2158479-supplement-MMC1.docx]

# **Supplementary Material**

**For: Children’s Susceptibility to Content Generated by Artificial Intelligence**

*Contents*

1. [Open-Ended Responses Coding Scheme](#_Open-Ended_Responses_Coding)
2. [Adult accuracy compared to child accuracy across stimulus category](#_Task_Accuracy_by)
   1. *Supplementary Figure 1.* Adult and child accuracy by stimulus category.
3. [Examples of the stimuli that were judged most and least accurately by child participants](#_Examples_of_the)
   1. *Supplementary Figure 2****:*** Scatterplots showing the 10 stimuli judged most and least accurately by all child participants
   2. Supplementary *Table 1****:*** Examples of stimuli with highest percentage of participants who answered correctly regarding the stimulus source (human or AI).

## Open-Ended Responses Coding Scheme

Coding implemented in RStudio using the *tidytext* package.

{r}

#-----------------------------

# Single-word dictionary (from actual responses)

#-----------------------------

heuristic_dictionary <- tibble(

word = c(

# Visual Quality Cues

"blurry", "blurred", "pixelated", "pixel",

"quality", "clear", "perfect", "background", "details", "design",

"stretched", "detail", "squeezed", "pixely", "colorful", "detailed", "color",

# Realism Judgments

"real", "fake", "realistic", "unrealistic",

# Content Plausibility

"wrong", "mistake", "sense", "fact", "facts", "true",

# Knowledge of AI

"ai", "computer", "generated", "smartbot", "Smarbot", "SmartBot",

# Anthropomorphic Reasoning

"person", "human", "people",

# Stimulus-Specific

"teacher", "school", "paint", "painted", "painting",

"picture", "photo", "book", "house",

# Uncertainty

"guess", "sure", "guessed"

),

heuristic = c(

rep("visual_quality", 17),

rep("realism", 4),

rep("content_plausibility", 6),

rep("knowledge_ai", 6),

rep("anthropomorphic", 3),

rep("stimulus_specific", 9),

rep("uncertainty", 3)

)

)

#-----------------------------

# Multi-word phrases

#-----------------------------

multiword_phrases <- tibble(

phrase = c(

# Visual Quality Cues

"too blurry", "looks blurry", "background is blurry",

"too perfect", "appears perfect", "looks perfect",

"can't see", "can't really see", "couldn't see", "don't see",

"close up", "it's close", "more solid",

"brush strokes", "brushstrokes", "brushstroke",

"short and quick", "not solid", "how wide it is", "because of how wide", "same size",

# Realism Judgments

"looks real", "looks like a real", "seems real",

"looks fake", "not real", "looks unreal",

"real photo", "real picture", "real image",

"like a real photograph", "looks painted",

"looks like she", "life-like", "real person",

# Content Plausibility

"doesn't make sense", "don't make sense", "makes sense",

"has mistakes", "mistake",

"facts are wrong", "wrong",

"don't come out", "doesn't come", "can't be",

"couldn't be", "wouldn't",

"same voice",

"said the name", "said 3 facts",

"more than 3 facts",

"this one felt true", "felt true",

"come from", "came from",

# Explicit Mention of AI

"smartbot", "ai generated", "ai made",

"smartbot makes", "smartbot could",

# Anthropomorphic Reasoning

"person took", "someone took", "person made",

"she could have", "he could have", "could have",

"could have gotten",

"she told", "he told",

"act the same", "acted",

"she probably", "he probably", "probably took",

"she might", "he might",

"good at", "creative",

"she likes", "he likes",

"asked people", "took pictures of",

"took a picture of", "picture of",

"would come from", "something that would",

"because she", "because he",

"she said it", "he said it",

"made it", "shelby made",

"she painted", "he painted", "she could paint",

"looks like she", "looks like he",

"took a long time", "ms shelby", "ms. shelby", "shelby", "a real person would",

# Stimulus-Specific

"art teacher", "is an art teacher",

"works at school", "at school",

"in ny", "in new york", "when you were",

"pigeons", "food", "eating",

"crayons", "painting", "painted it",

"in her house", "her house",

"book", "there is a book",

"lockers", "pictures of lockers",

"butterflies", "she likes butterflies",

"little bottles", "bottles",

# Uncertainty

"i think", "i guess", "i believe",

"not sure", "i'm not sure", "don't know", "i don't know",

"hard to tell", "really hard to tell",

"i'm guessing", "just guessing",

"don't really", "maybe"

),

heuristic = c(

rep("visual_quality", 21),

rep("realism", 14),

rep("content_plausibility", 20),

rep("knowledge_ai", 5),

rep("anthropomorphic", 42),

rep("stimulus_specific", 23),

rep("uncertainty", 13)

)

)

#-----------------------------

# Analysis

#-----------------------------

results <- analyze_heuristics(df, text_col = "text")

# Count how many responses use EACH category (can be multiple per response)

category_counts <- results %>%

summarise(

`Visual Quality Cues` = sum(visual_quality),

`Realism Judgments` = sum(realism),

`Content Plausibility` = sum(content_plausibility),

`Mentions of AI` = sum(knowledge_ai),

`Anthropomorphic Reasoning` = sum(anthropomorphic),

`Stimulus-Specific` = sum(stimulus_specific),

`Uncertainty` = sum(uncertainty),

) %>%

pivot_longer(everything(), names_to = "category", values_to = "count") %>%

arrange(desc(count)) %>%

mutate(prop = count / nrow(results))

#-----------------------------

# Count heuristics by judged_source

#-----------------------------

category_counts_by_source <- results %>%

group_by(judged_source) %>%

summarise(

`Visual Quality Cues` = sum(visual_quality),

`Realism Judgments` = sum(realism),

`Content Plausibility` = sum(content_plausibility),

`Mentions of AI` = sum(knowledge_ai),

`Anthropomorphic Reasoning` = sum(anthropomorphic),

`Stimulus-Specific` = sum(stimulus_specific),

`Uncertainty` = sum(uncertainty)

) %>%

pivot_longer(-judged_source, names_to = "category", values_to = "count") %>%

ungroup() %>%

# Order categories overall by total usage

group_by(category) %>%

mutate(total_count = sum(count)) %>%

ungroup() %>%

arrange(desc(total_count)) %>%

mutate(category = fct_reorder(category, total_count)) # Reorder by total usage

## Task Accuracy by Stimulus Category and Age Group


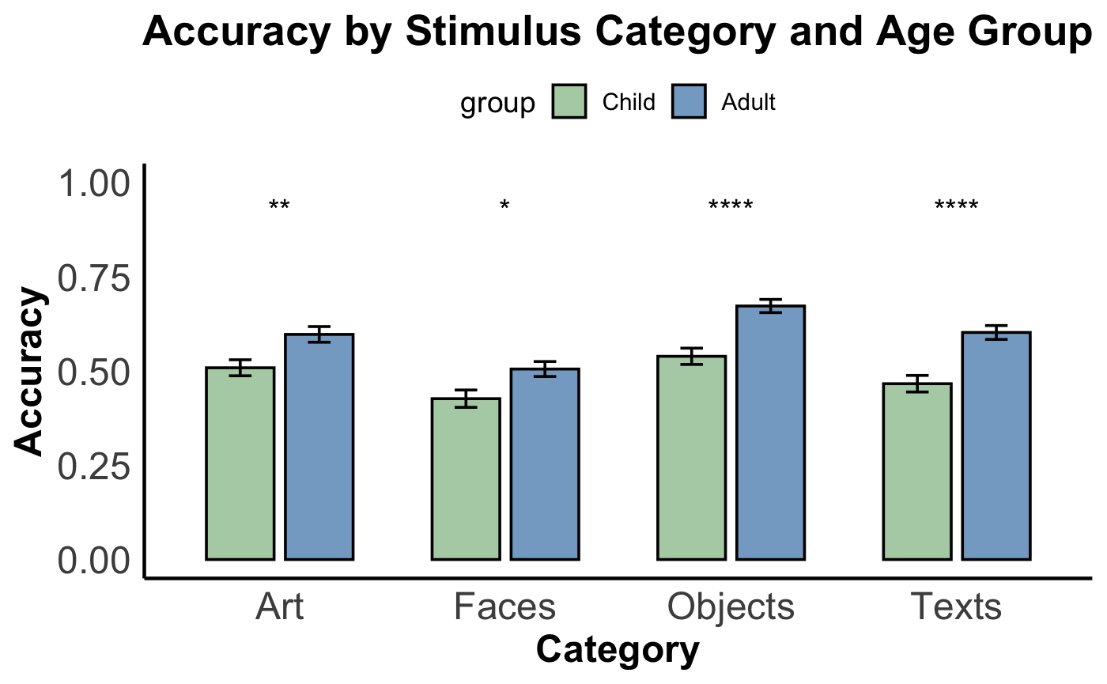


Supplementary Figure 1. Adult and child accuracy by stimulus category.

## Examples of the stimuli that were judged most and least accurately by child participants.


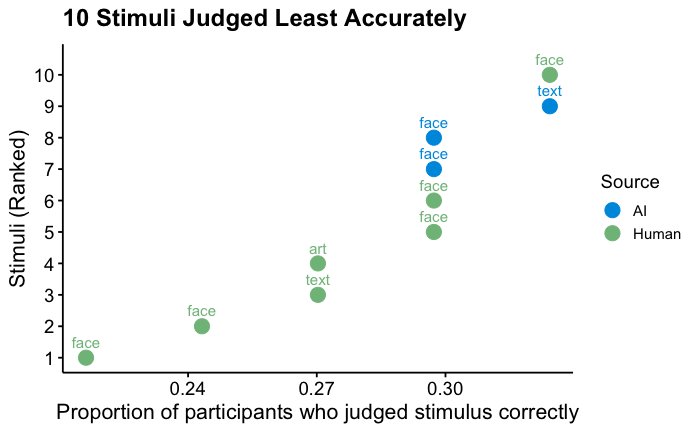

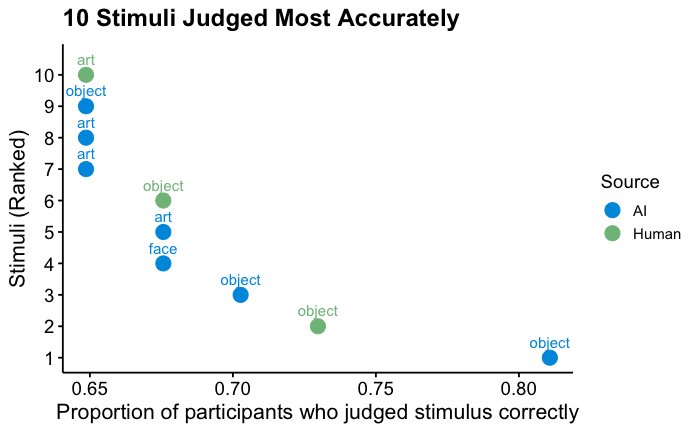


Supplementary Figure 2. Scatterplots showing the 10 stimuli judged least accurately by all participants (left) and most accurately by all participants (right), including the type of stimulus (faces, art, object, text), and its source (AI or human).

***Supplementary Table 2.*** *Stimuli with highest percentage of participants who answered correctly regarding the stimulus source (human or AI).*

| Source | Stimulus Category | % of participants who answered accurately | Stimulus |
| --- | --- | --- | --- |
| AI | Object | 81% | 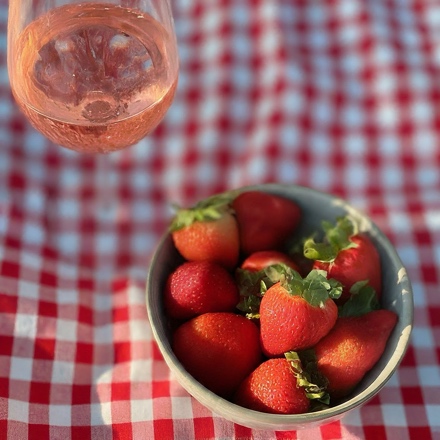 |
| Human | Object | 73% | 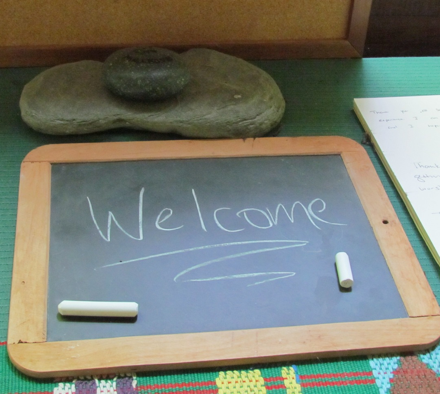 |
| AI | Object | 70% | 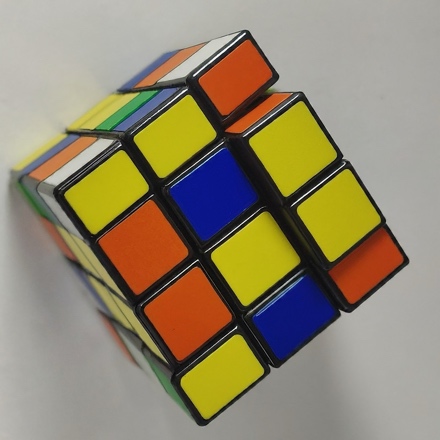 |
| AI | Art | 68% | 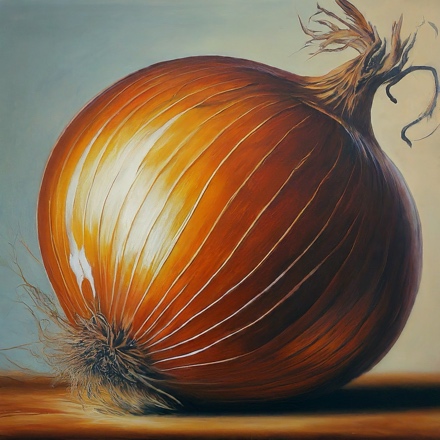 |
| AI | Face | 68% | 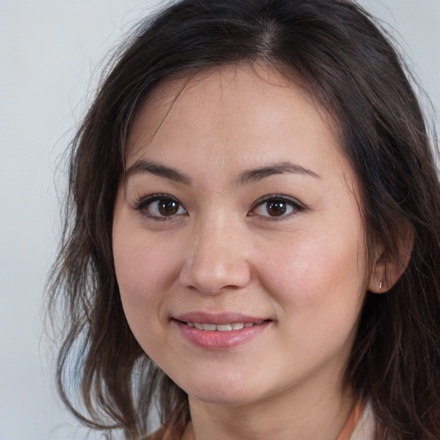 |
| Human | Object | 68% | 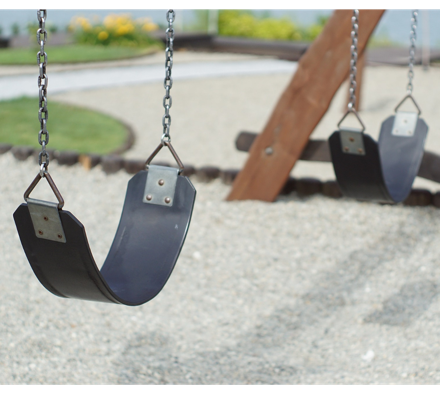 |
| AI | Art | 65% | 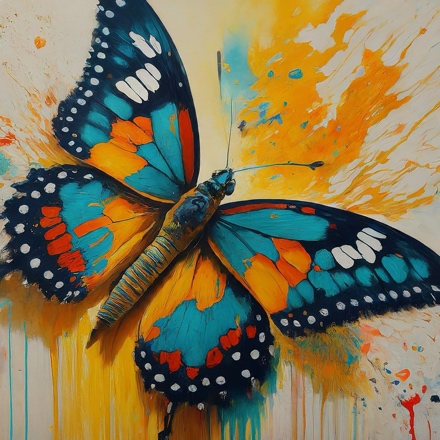 |
| AI | Art | 65% | 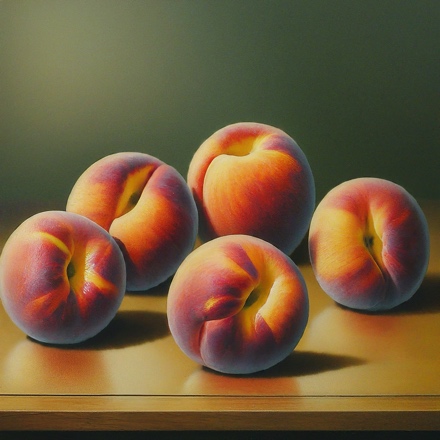 |
| AI | Object | 65% | 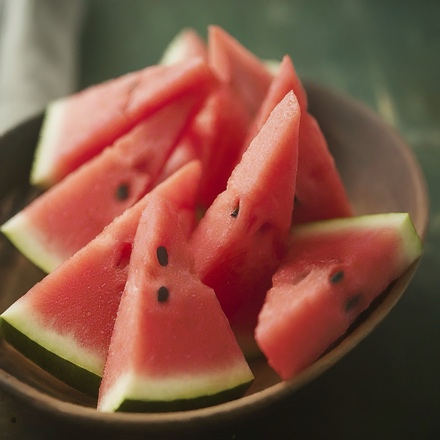 |

***Supplementary Table 2.*** *Stimuli with lowest percentage of participants who answered correctly regarding the stimulus source (human or AI).*

| Source | Stimulus Category | % of participants who answered correctly | Stimulus |
| --- | --- | --- | --- |
| Human | image | 22% | 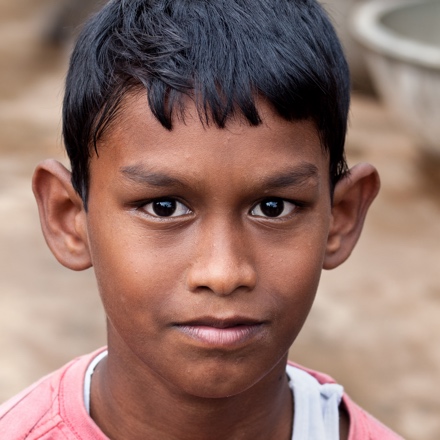 |
| Human | image | 24% | 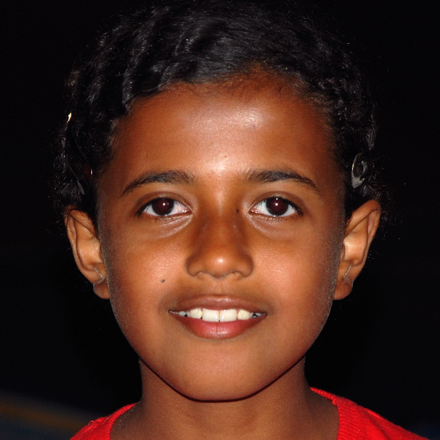 |
| Human | text | 27% | Earth is the only planet that has water on its surface. If Earth were closer to the sun, the oceans would boil aways. If it were farther away, the oceans would freeze. |
| Human | image | 27% | 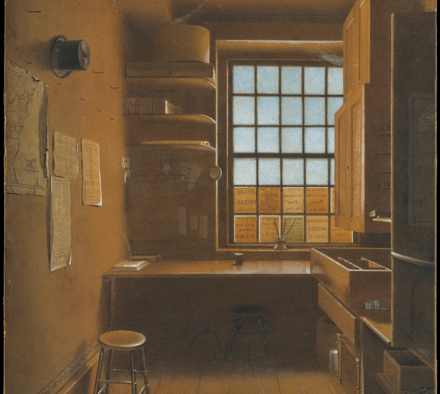 |
| AI | image | 30% | 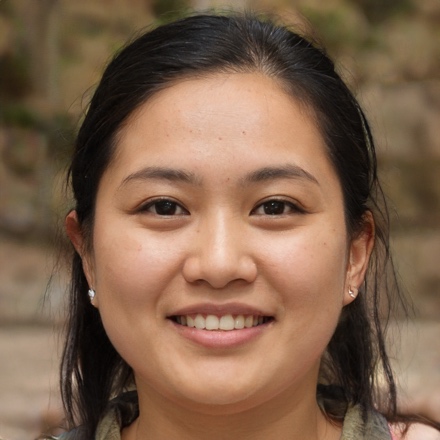 |
| AI | image | 30% | 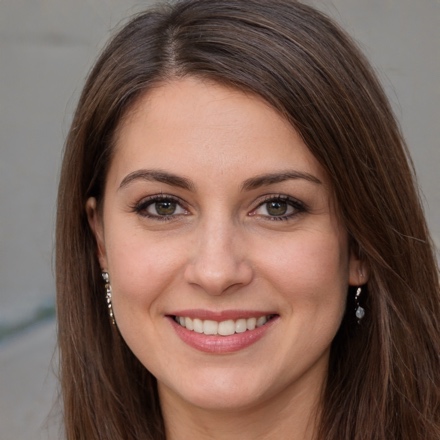 |
| Human | image | 30% | 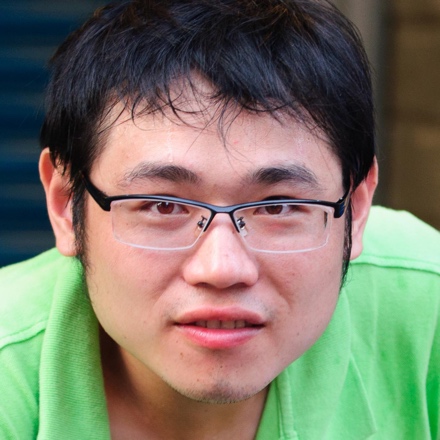 |
| Human | image | 30% | 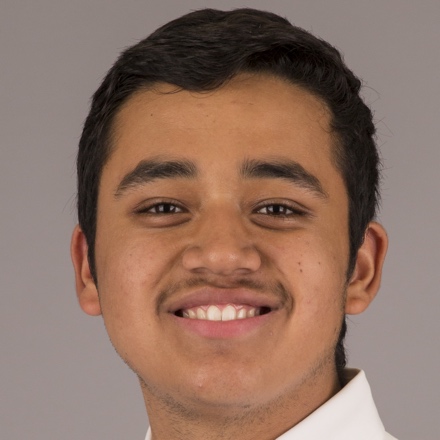 |
| AI | text | 32% | Summer at the beach is fun! We build sandcastles, splash in the waves, and collect pretty seashells. The sun is warm, and we can play all day in the sand and water. |
| AI | text | 32% | Butterflies are colorful insects with big, beautiful wings. They start as caterpillars and change into butterflies. They love to visit flowers and sip nectar, making gardens look magical and pretty as they flutter around. |
